# The Action Scales Model: A conceptual tool to identify key points for action within complex adaptive systems

**DOI:** 10.1177/17579139211006747

**Published:** 2021-05-15

**Authors:** James D Nobles, Duncan Radley, Oliver T Mytton, The Whole Systems Obesity programme team

**Affiliations:** The National Institute for Health Research Applied Research Collaboration West (NIHR ARC West), University Hospitals Bristol and Weston NHS Foundation Trust, Bristol, UK; Population Health Sciences, Bristol Medical School, University of Bristol, NIHR ARC West, 9th Floor, Whitefrairs, Lewins Mead, Bristol BS1 2NT, UK; Centre for Applied Obesity Research, Leeds Beckett University, Leeds, UK; MRC Epidemiology Unit, University of Cambridge, Cambridge, UK; Centre for Applied Obesity Research, Leeds Beckett University, Leeds, UK

**Keywords:** complexity, systems science, leverage points, complex intervention, health policy, complex adaptive systems

## Abstract

**Background:**

Systems thinking is integral to working effectively within complex systems, such as those which drive the current population levels of overweight and obesity. It is increasingly recognised that a systems approach – which corrals public, private, voluntary and community sector organisations to make their actions and efforts coherent – is necessary to address the complex drivers of obesity. Identifying, implementing and evaluating actions within complex adaptive systems is challenging, and may differ from previous approaches used in public health.

**Methods:**

Within this conceptual article, we present the Action Scales Model (ASM). The ASM is a simple tool to help policymakers, practitioners and evaluators to conceptualise, identify and appraise actions within complex adaptive systems. We developed this model using our collective expertise and experience in working with local government authority stakeholders on the Public Health England Whole Systems Obesity programme. It aligns with, and expands upon, previous models such as the Intervention Level Framework, the Iceberg Model and Donella Meadows’ 12 places to intervene within a system.

**Results:**

The ASM describes four levels (synonymous with leverage points) to intervene within a system, with deeper levels providing greater potential for changing how the system functions. Levels include events, structures, goals and beliefs. We also present how the ASM can be used to support practice and policy, and finish by highlighting its utility as an evaluative aid.

**Discussion:**

This practical tool was designed to support those working at the front line of systems change efforts, and while we use the population prevalence of obesity as an outcome of a complex adaptive system, the ASM and the associated principles can be applied to other issues. We hope that the ASM encourages people to think differently about the systems that they work within and to identify new and potentially more impactful opportunities to leverage change.

## Background

There is a rich and extensive history of systems science literature,^1^ but only somewhat recently has there been interest in the field of Public Health.^2,3^ Spearheading this interest, the UK Government Office for Science commissioned the ‘Tackling Obesities’ Foresight report.^4^ The report postulated that population level obesity was the product of a complex adaptive system, comprising an interconnected web of many causal factors. However, despite this, the uptake of systems approaches to address obesity and other public health challenges has been slow. A recent systematic review concluded that the application of systems approaches largely remains theoretical.^5^

Complex adaptive systems are characterised by several factors.^6,7^ They adapt over time in unpredictable ways in response to new policies, social norms, commercial interests and technological advancements for example. They are also characterised by interdependency and feedback; the component parts of the system influence one another, reinforcing or stabilising outcomes as they begin to emerge. For example, the increasing prevalence of convenience food reduces the need for people to cook. In turn, this reinforces the market demand for convenience food and leads to a greater supply, simultaneously, deskilling the population due to a reduced need to prepare and cook fresh meals. Other factors such as marketing of convenience food, the cost and availability of ingredients and the legislation around food policy all contribute to this complex interplay; an emergent property, among other things, being an increasing dependency on convenience food consumption. This example is nested within the overarching system that drives obesity at the population level.

It is now accepted that systems approaches should be adopted when aiming to fundamentally alter the obesogenic system.^4,5,8–10^ A systems approach aims to corral the public, private, voluntary and community sectors to make their actions and efforts coherent in a way that addresses the complexity of obesity^8,11^ – albeit that a formal definition is yet to be agreed upon.^5^ However, a current and pressing concern is that much of the work that is undertaken to prevent population level obesity does not take a systems approach. For example, Nobles et al.^12^ found that local government organisations were most likely to implement behaviour change programmes that encourage individuals to make healthier choices. Many of these interventions operate within a reductionist and medicalised paradigm (whereby interventions focus on specific elements of the system in isolation or aim to instigate change at the individual level rather than on populations and systems), are hypothesised to bring about predictable and consistent outcomes, and also assume that the context surrounding interventions will remain constant over time.^13^ Such interventions seldom account for the underlying, multifaceted nature of obesity (Table 1). The challenge now faced is how to re-orientate efforts to account for the complexity of the systems that we live and work within.

**Table 1:** Common features of reductionist and systems mindsets

	Reductionist mindset	Systems mindset
Purpose of action	… align with a reductionist paradigm. Action seeks to influence an isolated element of the system (if the system is acknowledged).	… understand that actions operate within a complex system, with the action seeking to influence how the system functions. Recognise that many coherent actions are required across the system. Difficult to isolate effect to individual actions.
Focus of action	… target specific causal factors (e.g. individual lifestyle behaviours).	… considers the patterns, structures and drivers which give rise to a system behaviour (i.e. the factors which cause a problem to occur).
Relationships between stakeholders	… likely to be transactional in nature whereby a provider is commissioned to deliver a specified service.	… understand that collaborative relationships and trust are imperative between stakeholders when seeking action.
Longevity of action	… anticipate that the system will remain static over time. The action will continue to create the same outcomes overtime and in different contexts.	… anticipate that the system is dynamic and adaptive, evolving over time in response to actions. Each complex problem is unique and therefore a shared understanding of the problem is required by involved persons. Actions will be highly context specific and dependent on the system boundaries.
Availability of an evidence base	… have an extensive empirical evidence base for discrete interventions. Often have well-funded research streams.	… may have a limited evidence base. Evidence may be more theoretical or hypothetical.
Evaluating action	… are easily measurable in isolation (e.g. have a number of key performance indicators). Indicators tend to be focused on the main outcome and the reach of interventions. Evaluation aims to prove effectiveness.	… assess impact in the context of the system. Determine whether the action is helping to change the functioning of the system in the anticipated direction. Focus on proxy measures of success. Aim to improve effectiveness.
	*Along a spectrum*	

Within systems theory, leverage points exist.^2,13–15^ These are modifiable points within a system that, if altered, can lead to changes in how the system functions.^14^ Identification of leverage points is deemed critical for achieving meaningful change, and practitioners and policymakers should aim to identify and modify these points within their own systems (which may lie within larger systems). Yet, moving from theory to practice is challenging. To move beyond this impasse, researchers have proposed tools to facilitate broader thinking about actions within complex systems.^14–16^ These tools include Meadows’ 12 places to intervene,^14^ the Intervention Level Framework (ILF)^16^ and the Iceberg Model.^15^

These tools have often been developed by researchers for researchers, which may make them difficult for people working in practice to utilise, given their dependency on systems science expertise. Consequently, we developed the Action Scales Model (ASM) to help practitioners and policymakers conceptualise, identify and appraise actions within a complex adaptive system. In doing so, it prompts people to think about, and identify, different leverage points and moves focus away from a reliance on traditional types of action (Table 1). Within this conceptual article, we aim to present the ASM and its component parts; explain the practical utility of the ASM; and illustrate how stakeholders can use the ASM to evaluate actions within a system.

## Methods

The ASM was created to sit within, and contribute towards, a larger body of work; the Whole Systems Obesity (WSO) programme.^17^ The aim of the WSO programme was to co-produce a guide, and an associated set of resources/tools, that enable local government authorities (LAs) in England to implement a whole systems approach to obesity. During the development of the WSO programme, we identified the need for a practical tool to help LA stakeholders think about and identify different types of actions, and the extent to which those actions may help leverage systems change. While other models are available to identify leverage points,^14–16^ formative assessment within the WSO programme (based on observation and discussion with LA stakeholders) suggested that these models were too abstract and complicated for real-world use (i.e. perceived as overly academic).

With this in mind, and taking on board the specific feedback from stakeholders during the development of the WSO programme, we developed a simpler tool that was relatively concrete. In doing this, we ensured that the new tool (the ASM) conceptually aligned with the three models aforementioned and retained some of the common features^14–16^ (see Table 2). We aimed to use terminology and visuals that would resonate with practitioners and policymakers, so that they could be used in local contexts related to practical issues and interventions.

**Table 2:** Leverage points – alignment between Meadows,^14^ Malhi et al.,^16^ Senge^15^ and the ASM

	Meadows’ 12 Points to Intervene	Intervention Level Framework	Iceberg Model	ASM
−Degree of leverage +	Power to transcend paradigms	Paradigm	Mental models	Beliefs
	Paradigm that the system arises out of			
	Goals of the system	Goals	System structures	Goals
	Power to add, change, evolve, or self-organise system structure	System structures		Structures
	Rules of the system			
	Structure of information flow			
	Gain around driving positive feedback loops	Feedback loops and delays	Patterns	
	Strength of negative feedback loops			
	Length of delays			
	Structure of material stocks and flows	Structural elements	Events	Events
	Size of buffers and other stabilising stocks			
	Constants, parameters and numbers			

ASM: Action Scales Model.

The alignment between the three models is not as distinct as presented here. For example, Malhi et al.^16^ suggest that ‘the rules of the system’ and ‘information flows’ may also be viewed as ‘structural elements’ if they relate to a particular sub-system or actor within the system.

We developed the ASM using our collective expertise and our experience of working closely with many LAs as part of the WSO programme. Core members of the WSO programme included applied health researchers, public health professionals and policymakers (local and national). The outputs of the WSO programme (a guide and complementary resources) were tested and refined by seven LAs, with the last iteration of the outputs being reviewed and approved by national and international experts. The results of the process evaluation related to the development of the WSO guide and resources is available elsewhere.^18^ The ASM formed one part of the WSO programme outputs.

The purpose of this conceptual article is to present the ASM to a wider audience, providing a more detailed account of its theoretical underpinnings and applicability than in the WSO programme outputs. We hope that this model encourages others to join the conversation around systems change efforts.

## Results

### The ASM

**Figure 1.**
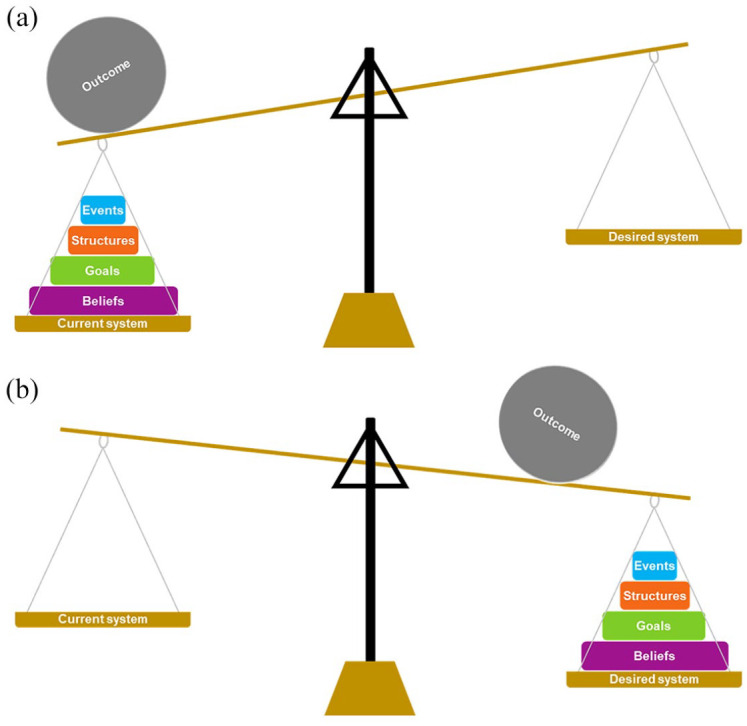
The Action Scales Model: (a) the current system which is imbalanced – for example, towards an obesogenic environment. It causes population weight to increase alongside compounding other issues associated with social inequality. The aim is for system architects (i.e. those who can influence how the system functions) to reorientate the system in a way which supports a healthier population weight (b). By leveraging actions deeper within the system (e.g. goals and beliefs), a tipping point is more likely to be reached which can cause rapid changes in the system structure to occur. Because goals and beliefs hold greater weight in the system, reorientating them towards a healthy weight system will require more effort than focusing on events.

The ASM aims to help enable practitioners and policymakers to both understand why the system functions as it does (which includes the people and organisations within it) and to identify opportunities to leverage change through action across the four levels. The ASM (Figure 1) is depicted as a set of scales, with the system made up of four levels: events, structures, goals and beliefs. Each level influences how the system functions, and in turn, the main outcome that the system produces (represented as the ball balanced atop the scales). In this article, the population prevalence of obesity could be regarded as the main outcome. The current system is configured in such a way that it promotes population level obesity – referred to as the obesogenic system.^19^

When seeking to understand the system functioning, *events* relate to the issues (behaviours and proxy outcomes) that can be observed around us and are symptoms of the system working as designed (both intentionally and unintentionally). For example, convenience food is readily available and widely consumed, cars are the dominant mode of transport, and workplaces observe high levels of presenteeism and absenteeism. *Structures* relate to the patterns, relationships, information flows and physical structures that cause events to occur. Related to the example above, transport infrastructure is predominantly designed to support car use, from the development and sustainment of road transport networks to the design and layout of new housing developments. *Goals* refer to the ambitions or targets that the system (or parts within the system) are working towards. These goals influence how the system is structured, and therefore, how it functions and the outcomes it produces. It is also important to differentiate between the stated goals (i.e. those *said* to be working towards) and observable goals (i.e. those *being* worked towards); a discordance is often present. A workplace, for example, may state that employee wellbeing is a key organisational priority but does little to change the organisational structures to influence staff wellbeing. Finally, it is the *beliefs*, norms, values and attitudes of systems architects (i.e. those who influence the structure and workings of a system) that cause the system to function as it does. People who hold power within a system can influence how resources are distributed and decisions are made across the system.^13^ A senior executive within an organisation may believe that the sole purpose of the workplace is to generate revenue. In turn, this would influence the goals of the system (e.g. for employees to meet sales targets), which then dictates how the organisation is structured and how resources are managed, which then impacts its revenue. As can be seen, the four levels are interconnected.

The ASM has been designed to support practitioners and policymakers to identify leverage points in order to change how the system functions. The four levels within the ASM are graphically depicted as weights; the larger the weight, the greater the likelihood of leveraging systems change. In the context of obesity, many actions are currently implemented at the *event*-level (e.g. educating people about high-sugar drinks, provision of weight management programmes, implementation of the Daily Mile in schools). These are reactive actions, often thought of as quick fixes. They offer little leverage for system change and do little to reduce the likelihood of the event recurring in the future, hence why they are the smallest weight within the ASM. They are also likely to be the easiest to implement from resource, political and evaluative perspectives. At the *structure*-level, actions offer more leverage because they aim to reduce the likelihood of events happening again in the future (i.e. the patterns of an event) by anticipating where and how issues may arise. They seek to alter the physical (i.e. built or natural infrastructure), relational (i.e. the relationships and rules between the parts of the system and the actors within it) and informational (i.e. how information flows between parts of the system and the actors within it) structures known to be driving the problem, and thus necessitate a thorough understanding of the system (Table 2). They work to interrupt the relationships between the parts of the system, relationships which may form positive or negative feedback loops that reinforce the recurrence of a problem (refer back to convenience food example above). Actions targeting the *goals* and *beliefs* require fundamental alterations to the system and the way in which things are done – they seek a paradigm shift and to change the status quo. To do so, change efforts must seek to influence the system architects and dominant beliefs, but the mechanisms by which this is achieved will vary greatly. These levels offer the greatest leverage for change, as depicted by their size (see Figure 1), but will likely be the most difficult to change. Table 3 summarises this information and provides examples of action at each level.

**Table 3:** Examples of coherent actions across the ASM

	Events	Structures	Goals	Beliefs
What we observe	These are the issues (behaviours and outcomes) that can be observed around us in the modern world, and are symptoms which arise from the system functioning as designed (both intentionally and unintentionally).	This relates to the underlying structures and patterns that cause the events to occur. This includes the organisation of the system; the structures, information flows, processes and relationships between parts of the system.	These are the goals, targets or ambitions that the system – or parts of the system – is working to achieve. Goals often drive the system to be structured as it is and therefore to work as it does.	These are the deeply held beliefs, norms, attitudes and values (i.e. the mindset) of the individuals and organisations within the system. They are the foundations that cause the system to keep functioning as it does, and are reflected in the system goals.
Actions at this level	Aim to suppress the *immediate* event. They do this by reacting quickly to the visible issues – i.e. ‘quick fixes’. Quite often these actions are needed, but will not address the underlying issues which cause the issue to arise (i.e. the structures, goals and beliefs).	Aim to reduce the number or severity of the events occurring. They do this by reshaping or redesigning the organisational or relational system structures, and therefore require an understanding for how the system works.	Aim to re-orientate the goals that the system is working towards. They do this by changing the beliefs of those people setting the system goals.	Aim to change how individuals and organisations (who influence how the system works) think about the problem. They do this by challenging and changing the deeply held beliefs, norms, attitudes and values within the system.
Example actions	1.1. LAs provide cycling training to school children.	1.2. The LA assesses and improves the walkability of the environment surrounding the schools.	1.3. Schools work with parents and community to set a shared goal to reduce short car journeys to school by 20% in next 5 years.	1.4. LA creates a working group to champion and promote active transport to senior leaders in the council.
	2.1. Families can attend free workshops to learn how to cook healthy food.	2.2. Regulations are introduced that require food manufacturers to reformulate ready meals.	2.3. Supermarket chains set mandatory targets for suppliers on the nutritional quality of products.	2.4. Supermarkets work with suppliers to demonstrate that healthier food options can maintain company profits.
	3.1. GPs refer adults with obesity to commercial weight management programmes.	3.2. Medical students receive mandatory training about the complexity of obesity.	3.3. Ensure that everyone, regardless of their health status, has access to a GP within one week.	3.4. Senior clinicians reinforce across healthcare settings that obesity is the product of complex adaptive systems.
Evaluating actions using the ASM^a^	*Using 1.1 as an example*: LA assess the cycling self-efficacy of school children. Also able to monitor the number of trips to school via active transport. May also monitor wider impacts on child’s educational attainment and engagement in class.	*Using 2.2 as an example*: Audit the alterations made to food regulations, and assess the impact of these regulations on (a) nutritional quality of products and (b) purchasing patterns of consumers. Ensure that unintended consequences are captured.	*Using 3.3 as an example*: Evaluate the number of people accessing their GP within 1 week. Assess the impact of this policy on patients, GPs, healthcare managers and administrative staff. Analyse patient data to understand equity of care.	*Using 1.4 as an example*: Interview senior leaders in the LA to determine their beliefs towards active transport, and whether these beliefs have changed due to intervention effort. Examine voting patterns of councillors with regards to active transport proposals.

ASM: Action Scales Model; LA: local government authority.

a When evaluating actions within a system, evaluators must ensure that they evaluate the collective impact of the actions, and the implications of these actions on their interdependencies (i.e. the parts of the system that may also be affected by these actions).

Given the interconnectivity between the levels of the ASM, it is important to think about their *collective coherence* (i.e. the extent to which events, structures, goals and beliefs in the system reinforce one another).^8^ The concept of coherence is applicable when aiming to understand the system and when identifying opportunities to intervene. To maximise the likelihood of systems change occurring, stakeholders should seek to intervene across *multiple* levels of the ASM, and in doing so, ensure that their efforts are mutually reinforcing. For example, implementing a 20-mph speed limit in a residential area may be best achieved by the following set of actions: (a) stakeholders promoting the benefits of the restriction to elected members and the public (i.e. targeting the system beliefs); (b) changing the goals of the system, from prioritising the speed of through traffic to the safety of local residents and the walkability of the local environment; alongside (c) creating the structures to directly implement the policy change (event or structural level). If the beliefs and goals are assessed and targeted prior to structural changes occurring, then this may increase the impact and sustainability of a change effort, and indeed, would make structural changes easier to implement. Additional examples of coherent actions are provided in Table 3.

### Using the ASM in practice and policy

The ASM has three primary uses for stakeholders working in practice and policy: (a) to help understand how the system works, and explain why the system generates the outcomes it does, (b) to facilitate the identification of leverage points for systems change, and (c) to ensure that there is coherence among actions being implemented and/or planned.

There are many ways to create a shared understanding of how a system functions, from systems mapping, to producing causal loop diagrams, to root cause analysis, to the development of rich pictures.^13,20,21^ As a minimum, the ASM can facilitate multistakeholder conversations to stimulate deeper thinking about a complex issue. Soft-systems methodologies acknowledge that the process of engaging in such conversations, and in understanding the different perspectives held between stakeholders, is more important than seeking an objective reality.^13^ If stakeholders have developed a systems map, or similar (e.g. a concept map, a causal loop diagram, an agent map), the ASM can be used to critically think about the causes (of obesity) included within the map; to reflect on why the system functions as it does, the level at which the causes operate, and the extent to which causes are interconnected. Whether in conversations or through systems maps, questions should be posed that cause stakeholders to reflect on the structures, goals and beliefs which cause the events to occur within the system (Table 4); these should also consider the social, political and cultural aspects of the system. The ASM facilitates stakeholders in acquiring a deeper understanding of the system. Importantly, however, the emphasis of the conversation should be placed on thinking broadly, and differently, about the system and considering all levels of the ASM, rather than on the correct classification of causes against one of the four levels. By obtaining a better understanding of how the system functions, and why it functions as it does, it then becomes possible to think more broadly about actions to intervene within the system.

**Table 4:** Questions which can be used to understand system functioning

ASM level	Questions
Event	(a) What issues or problems keep arising despite efforts to rectify them? (b) Where are intervention efforts targeted? Do they tend to focus on those that are affected by the problem? (c) Are the actions likely to stop the problem reoccurring in the future? (d) Do the actions seek to generate outcomes quickly and are they unlikely to be opposed by systems architects?
Structures	(a) What elements make up the system? Consider physical structures, people and organisations, interconnections and relationships, and information that flows between the elements of the system. (b) How are these elements organised or arranged? (c) Which of these elements cause the problems or events to occur? Also consider the connections between the elements. (d) What is the nature of the relationships between elements in the system? Do they self-regulate (i.e. one increases, the other decreases) or do they self-reinforce (i.e. one increases, the other increases)? How long does it take for these changes to occur? (e) Who has access to information about the system, and the elements within the system?
Goals	(a) What are the system/organisations/key individuals aiming to achieve within their spheres of influence? (b) What purpose do these systems/organisations/individuals hope to serve? (c) How are the system structures organised and why are they organised in this way? (d) Do the goals of multiple systems influencers overlap? To what extent could they be aligned? (e) Are the goals of the system currently supported by actions?
Beliefs	(a) What are the prevailing assumptions, beliefs and values that explain why things are done as they are? (b) Who (people and organisations) are the key decision makers within the system? What values, perspectives and priorities do they hold? (c) To what extent do these key decision makers believe that change is necessary, feasible and/or desirable? (d) What beliefs do these people and organisations hold regarding how the system works, and the goals that the system is working towards? (e) What is of fundamental importance to these people and/or organisations? (f) What are the beliefs of others who may be affected by systems change? Do they support or oppose the dominant belief within the system or the goals that it is working towards?

ASM: Action Scales Model.

When stakeholders have identified a part of the system that they, as a collective or individually, can influence using their expertise, resources or networks, the ASM provides a framework to help understand where to intervene within the system to maximise the likelihood for greatest leverage (i.e. at what level of the ASM). Within the WSO programme, the team developed an action planning tool to help stakeholders identify actions and to ensure coherence between them.^17^ This tool, taken in conjunction with the ASM, provides a structured approach to generating a coherent action plan. By this, we mean that actions are mutually reinforcing; that they work towards the same outcome and efficiently use available resources. The outcome does not necessarily need to be changing the prevalence of obesity, but may be more proximal such as improving the quality of food within newly established fast food outlets or enhancing the cohesion between multisectoral stakeholders. To remain efficient, stakeholders should seek to understand what actions are already underway within their system as well as considering how new actions may be introduced – all of which can be considered through the lens of the ASM. At all times, it is important that stakeholders focus on the part(s) of the system that they can influence to avoid becoming overwhelmed by the complexity of the system, and consequently disengaging in the process. Example scenarios are provided in Table 3 with regard to actions within systems.

Remaining pragmatic is important when using the ASM. Obesity, as with other complex issues, is often politically entangled in financially constrained contexts; for those working in public health, there is often a need to demonstrate tangible outcomes in short timescales, while working towards a longer term vision or strategy.^22–24^ Such pressures have previously led to a focus on downstream interventions, commissioned by siloed and fragmented bodies, with an intention to demonstrate return on investment.^12^ A systems approach aims to fuse these fragmented bodies together through collective, complementary and mutually beneficial agendas to make efficient use of available resources. Acknowledging this, the ASM should challenge multisectoral stakeholders to look deeper into the system, to identify other opportunities to leverage systems change, and to improve the coherence between their systems change efforts.

Achieving systems change will require a substantial amount of time and sustained effort,^8,25^ and thus, actions should be taken which are both episodic and continuous.^13^ Episodic actions are planned, time-limited and seek incremental improvement – often operating at the event and structural levels of the ASM. Simultaneously, continuous efforts are needed to address the underlying goals and beliefs held within the system – the systemic root causes of a problem. The development of an agile monitoring framework, which includes a range of metrics that allow the system functioning to be regularly monitored, will help stakeholders to demonstrate progress towards the long-term vision (rather than a reliance on ‘quick wins’). That said, stakeholders should not be overly reactive to contradictory or negative findings within their monitoring framework; within systems, things may worsen before they improve. Similarly, it is important to note that quick wins do serve a function in maintaining stakeholder enthusiasm in such an approach.

### Using the ASM to guide evaluation

Public health actions and interventions are traditionally monitored via key performance indicators and outcome measures, with success often being defined as the reach of an intervention and the extent to which an intervention brings about a notable change in the main outcome (see Table 3). Measuring change within complex adaptive systems is perhaps more challenging; it acknowledges that changes to the main outcome (e.g. obesity) will occur when the system, and the parts of the system, are fundamentally reorganised.^3^ However, outcomes which are the product of a complex adaptive system are unlikely to change quickly, and are very unlikely to change in response to single interventions.^3,8,26^ As such, determining the success of an intervention based upon its ability to influence the prevalence of obesity is misplaced; the focus should instead be upon whether the action contributes to a change within the system.^3^ A movement from the study of attribution to contribution, the ASM can be used to understand how and where such changes may have occurred across the various levels of the system, from events through to beliefs. Below, we outline several ways in which the ASM can be used to support evaluation efforts.

First, evaluators should understand the systems which are targeted by intervention efforts.^3,21,27,28^ As aforementioned, methods such as systems mapping can be used to visualise the system, and evaluators can then use models such as the ASM to understand the factors which drive the system. Evaluators can also adopt the same approach to analyse intervention efforts. As an example for how this may work, Nobles et al.^12^ applied the Wider Determinants of Health model to evaluate local government organisation efforts to prevent and treat population level overweight and obesity in the context of the local causes of obesity. This encourages local policymakers and practitioners to reflect upon their current approaches to obesity. The ASM could feasibly be used in place of the Wider Determinants of Health model. Other models akin to the ASM (e.g. the ILF)^16^ have been used in a similar manner^2,10,16,29^ to evaluate actions and policies on food/obesity systems,^2,16^ the social determinants of health^10^ and otitis media middle ear disease.^29^ As such, these models provide useful frameworks by which to analyse intervention efforts within complex adaptive systems.

Second, several research groups have suggested that qualitative methods can be used to evaluate systems change efforts. For example, Egan et al.^21^ highlight that ‘qualitative research with a systems lens’ is an accessible way to evaluate systems approaches, or aspects of one. They suggest that interview questions, may for example, aim to understand the different perspectives of various stakeholders, assess the intended and unintended consequences of implementation efforts, or determine the emergent and self-organisational properties as systems change occurs. We would add that evaluators can frame interview questions around the ASM. We have compiled a list of questions that can be used to help understand how the system functions, and subsequently, how actions may work within these systems (Table 4). In a similar vein, the ASM can then guide a deductive analytical framework.

Third, the ASM can help evaluators to identify proximal and intermediate outcomes to focus upon. Given that the main outcomes of complex adaptive systems (e.g. population levels of obesity) are unlikely to change within a short timeframe, proxy indicators are needed to help determine whether intervention efforts are bringing about favourable changes in the system.^3^ This information can be of great importance to stakeholders with a vested interest in the intervention. For example, if systems change efforts were being implemented to increase the number of families walking their children to school (i.e. the main, long-term outcome), then using the ASM, evaluators may wish to collect data on the quality of active travel infrastructure surrounding schools and the presence of cycle storage at schools (i.e. structures). They may also wish to monitor the explicit goals that local stakeholders are working towards, for example, those which are written in key documents published by schools and local government organisations. Again, the ASM would then provide the analytical framework to create a coherent evaluation narrative for a system change effort such as this.

Finally, evaluation designs such as comparative case analysis can create compelling accounts for how a system may have changed over time. These designs take a mixed-methods approach (e.g. using informant interviews, social network analysis, epidemiological analysis) to describe the current state of the system, and then repeat this approach after a given time frame, to describe the features and workings of the new system. Matheson et al.^30^ provide a good example of this design in the context of a community-based public health intervention in New Zealand. These comparative case designs can be guided by models such as the ASM, both from the viewpoint of data collection (i.e. to guide interview questions as aforementioned) and from an analytical standpoint. However, given that these designs use mixed methods, the ASM can provide an underpinning theoretical model to triangulate and synthesise research findings.

## Discussion

### Comparison to other tools

The ASM has several similarities with the other available models – the ILF,^16^ the Iceberg Model^15^ and Meadows’ 12 places to intervene ^14^ (Table 2). First, each model aims to stimulate broader thinking about actions within systems. They are hierarchical models which stipulate that certain types of actions (e.g. mental models (Iceberg), paradigms (ILF) and system beliefs (ASM)) hold more leverage than others for systems change. The models, including the ASM, also outline that the greatest leverage will come about when there is coherence between actions at the respective levels. Re-orientating the beliefs (or mental models (Iceberg)) held within the system should then enable the goals of the system to be changed, and the structures within the system can then be altered accordingly. Each model therefore posits that actions should target multiple levels of the system simultaneously, to create a collective effort in the same direction. Finally, the ILF, the Iceberg Model and the ASM correspond with the 12 places to intervene,^14^ and given Donella Meadows’ prominence in the field of systems science, alignment with her work adds credibility and robustness to the simplified models.

However, the simplification of Meadows’ work also means that our model, the ILF, and the Iceberg Model are less nuanced. These models group several of Meadows’ 12 leverage points together and categorise them into one of four or five levels (see Table 2). In doing so, these models limit the opportunity for discussion around the omitted points of feedback loops, the length of delays, stocks and flows and so on. Consideration though needs to be given to practical utility of a model. If a 12-item model was to be used to identify leverage points, how feasible would it be for stakeholders to use it? Illustrating this point, the ILF was devised to improve the coding of qualitative survey data when evaluating actions within systems, as interrater reliability was poor when attempting to apply Meadows’ list.^16^ For the ASM, the objective was to create an understandable model that could be applied by practitioners and policymakers. Our scoping work in the WSO programme would suggest that the adoption of Meadows’ list of leverage points would require substantial systems science expertise, therefore making it unsuitable for these purposes. We also considered the depiction of the model; in representing it as a set of scales and weights, users can see the leverage held by the various levels of the ASM which differentiates it from the ILF and Meadows’ 12 places to intervene.

### Strengths and limitations of the ASM

The ASM was created in response to the challenges of *applying* existing models (Table 2) in local contexts by practitioners and policymakers. For example, the Iceberg Model^15^ is used predominantly within the private sector to facilitate change management efforts, and the ILF^16^ has been adopted by researchers in the public health field to evaluate interventions, policies and systems change.^2,10,29,31^ We hope that the ASM can be used for several purposes: to understand why the system works as it does, to identify and subsequently appraise actions and finally, to guide elements of an evaluation. Thus, the purpose and use of the ASM differs from previous models in that its application is broader, but due to the simplicity of the model and the ease of understanding, it can be applied without the expertise of a third party. Although not yet applied in another context, we also believe that the model can be used to better understand other problems that are entwined with complex adaptive systems.

As with other models, and as noted previously, there are limitations to the ASM. The model was developed to sit within the WSO programme. The WSO programme provides a framework by which stakeholders can consider how they may work as a collective to implement a systems approach. This broader work introduces some of the systems science theory. Knowledge of this is anticipated to support stakeholders as they progress through the WSO framework. As such, the application of the ASM in isolation of this wider work is likely to be more challenging than if used alongside it. Similarly, whilst use of the ASM is not hinged upon the presence of a systems map, we do believe that these visual depictions will help stakeholders to use the ASM as they promote a collective understanding of a complex problem. In absence of a systems map, the ASM is still likely to be useful in thinking more critically, and systemically, about challenges currently being faced. Aligned with the two points above, some training may still be required to use the ASM – particularly if it is not being used as part of the wider resources within the WSO programme.

## Conclusion

The calls to adopt systems approaches within fields of public health and healthcare have grown substantially in recent years. Given the complexity of the challenges we face in the 21st century, linear and reductionist ways of working are insufficient. Systems approaches are needed but are difficult to implement. We have presented a novel tool to help stakeholders to explore how the system is currently functioning, to question why some of the issues may be arising, and finally, to identify where and how to intervene in the system. It can also serve as a mechanism to bring together cross-sectoral stakeholders in order to reflect on current practice, and to think broadly about the future approach. Finally, we see that the ASM can be used as a tool to guide evaluation. The ASM will hopefully enable stakeholders to create a coherent approach which may bring about greater systems change.

## References

[1] Castellini B. Theory, Culture & Society 2014. Available online at: https://www.theoryculturesociety.org/brian-castellani-on-the-complexity-sciences/

[2] Johnston LM Matteson CL Finegood DT. Systems science and obesity policy: a novel framework for analyzing and rethinking population-level planning. Am J Public Health 2014;104(7):1270–8. 24832406 10.2105/AJPH.2014.301884 PMC4056198

[3] Rutter H Savona N Glonti K , et al The need for a complex systems model of evidence for public health. Lancet 2017;390(10112):2602–4. 28622953 10.1016/S0140-6736(17)31267-9

[4] Butland B Jebb S Kopelman P , et al Tackling obesities: future choices: project report (ed Foresight). London: Government Office for Science; 2007. p. 164. 10.1111/j.1467-789X.2007.00344.x 17316292

[5] Bagnall A-M Radley D Jones R , et al Whole systems approaches to obesity and other complex public health challenges: a systematic review. BMC Publ Healt 2019;19(1):8. 10.1186/s12889-018-6274-z PMC6318991 30606173

[6] Finegood DT. The complex systems science of obesity. In: Cawley J (ed.) The Oxford Handbook of the social science of obesity. New York: Oxford University Press; 2011.

[7] Shiell A Hawe P Gold L. Complex interventions or complex systems? Implications for health economic evaluation. BMJ 2008;336(7656):1281–3. 18535071 10.1136/bmj.39569.510521.AD PMC2413333

[8] Swinburn BA Kraak VI Allender S , et al The global syndemic of obesity, undernutrition, and climate change: *The Lancet* Commission report. Lancet 2019;393(10173):791–846. 30700377 10.1016/S0140-6736(18)32822-8

[9] Capewell S Capewell A. An effectiveness hierarchy of preventive interventions: neglected paradigm or self-evident truth? J Publ Healt 2018;40(2):350–8. 10.1093/pubmed/fdx055 28525612

[10] Carey G Crammond B. Systems change for the social determinants of health. BMC Publ Healt 2015;15(1):662. 10.1186/s12889-015-1979-8 PMC4501117 26168785

[11] House of Commons Health Committee. Childhood obesity: time for action – eighth report of session 2017–19. London: House of Commons; 2018.

[12] Nobles J Christensen A Butler M , et al Understanding how local authorities in England address obesity: a wider determinants of health perspective. Healt Pol 2019;123(10):998–1003. 10.1016/j.healthpol.2019.07.016 31431294

[13] Foster-Fishman PG Nowell B Yang H. Putting the system back into systems change: a framework for understanding and changing organizational and community systems. Am J Community Psychol 2007;39(3–4): 197–215. 17510791 10.1007/s10464-007-9109-0

[14] Meadows D Wright D. Thinking in systems: a primer. Hartford, VT: Chelsea Green Publishing; 2008.

[15] Senge PM. The fifth discipline: the art and practice of the learning organization. New York: Doubleday/Currency; 1990.

[16] Malhi L Karanfil O Merth T , et al Places to intervene to make complex food systems more healthy, green, fair, and affordable. J Hunger Environ Nutr 2009;4(3–4): 466–76. 23173029 10.1080/19320240903346448 PMC3489112

[17] Public Health England. Whole systems approach to obesity programme: a guide to support local approaches to promoting healthy weight. London: PHE Publications; 2019.

[18] Public Health England. Whole systems approach to obesity programme: learning from co-producing and testing the guide and resources. London: PHE Publications; 2019.

[19] Swinburn B Egger G. Preventive strategies against weight gain and obesity. Obes Rev 2002;3(4):289–301. 12458974 10.1046/j.1467-789x.2002.00082.x

[20] Egan M McGill E Penney T , et al Complex systems for evaluation of public health interventions: a critical review. Lancet 2018;392: S31.

[21] Egan M McGill E Penney T , et al NIHR SPHR guidance on systems approaches to local public health evaluation – part 2: what to consider when planning a systems evaluation. London: National Institute for Health Research School for Public Health Research; 2019.

[22] Allender S Brown AD Bolton KA , et al Translating systems thinking into practice for community action on childhood obesity. Obes Rev 2019;20(Suppl. 2):179–84. 31359617 10.1111/obr.12865 PMC6900082

[23] Orton LC Lloyd-Williams F Taylor-Robinson DC , et al Prioritising public health: a qualitative study of decision making to reduce health inequalities. BMC Publ Healt 2011;11(1):821. 10.1186/1471-2458-11-821 PMC3206485 22014291

[24] Van de Goor I Hämäläinen RM Syed A , et al Determinants of evidence use in public health policy making: results from a study across six EU countries. Healt Pol 2017;121(3):273–81. 10.1016/j.healthpol.2017.01.003 PMC5754321 28139253

[25] Davies S Pearson-Studdard J. Tackling childhood obesity: evidence, persistence and political will. BMJ Opin 2018;361:k2775.

[26] Swinburn BA Sacks G Hall KD , et al The global obesity pandemic: shaped by global drivers and local environments. Lancet 2011;378(9793):804–14. 21872749 10.1016/S0140-6736(11)60813-1

[27] Egan M McGill E Penney T , et al NIHR SPHR guidance on systems approaches to local public health evaluation – part 1: introducing systems thinking. London: National Institute for Health Research School for Public Health Research; 2019.

[28] Moore GF Evans RE Hawkins J , et al From complex social interventions to interventions in complex social systems: future directions and unresolved questions for intervention development and evaluation. Evaluation 2018;25(1):23–45. 30705608 10.1177/1356389018803219 PMC6330692

[29] Durham J Schubert L Vaughan L , et al Using systems thinking and the intervention level framework to analyse public health planning for complex problems: Otitis media in Aboriginal and Torres Strait Islander children. PLoS ONE 2018;13(3):e0194275. 29561891 10.1371/journal.pone.0194275 PMC5862467

[30] Matheson A Walton M Gray R , et al Evaluating a community-based public health intervention using a complex systems approach. J Publ Healt 2017;40(3):606–13. 10.1093/pubmed/fdx117 28977467

[31] McIsaac JD Spencer R Stewart M , et al Understanding system-level intervention points to support school food and nutrition policy implementation in Nova Scotia, Canada. Int J Environ Res Publ Healt 2019;16(5):172. 10.3390/ijerph16050712 PMC6427736 30818856

